# Characterization of goat rumen-derived isolates and their effects on *in vitro* ruminal fermentation properties

**DOI:** 10.3389/fvets.2026.1877756

**Published:** 2026-07-03

**Authors:** Yushu Zhang, Yutaka Uyeno

**Affiliations:** Graduate School of Science and Technology, Shinshu University, Nagano, Japan

**Keywords:** bacterial community, functional analysis, goats, lactic acid-producing bacteria, probioitcs

## Abstract

The goat rumen bacterial community is a potential source of probiotics, the impact of which on the microbial community when administered is unclear. Four isolates obtained from adult goat rumens were identified as *Enterococcus* sp. (isolate 11), *Streptococcus* sp. (isolates 14 and 7), and *Pediococcus* sp. (isolate B). A batch culture system was used to evaluate the effects of these four isolates at three inoculation levels on *in vitro* rumen fermentation characteristics, rumen microbial community composition, and predicted functional profiles. Compared to the control fermentation, the inclusion of any isolate reduced the pH, acetate proportion, and acetate-to-propionate ratio, while increasing the total volatile fatty acid (VFA) concentration and propionate proportion (PA%). Isolates 11 and 14 increased total gas production compared to the control, whereas Isolate B showed the lowest pH, highest PA%, and total VFA concentration. The microbial community structure was strongly influenced by the inclusions. Functional prediction of the community determined differential pathways related to metabolic activity. In particular, isolate B (*Pediococcus* sp.) yielded the highest relative abundance of *Xylanibacter* and *Prevotella*, ultimate carbohydrate-metabolizing members toward a greater representation, may have enhanced the predicted functional potential for the metabolism of multiple sugars, resulting in increased VFA production. Isolates 11 (*Enterococcus* sp.) and 14 (*Streptococcus* sp.) were mainly associated with enhanced fermentation activity and showed enrichment in fiber-metabolizing bacteria compared to the inclusion of other isolates. Although the supplementary effect on specific bacterial taxa was minor, the inclusion of isolates enriched different predicted pathways and showed a convergent potential for enhancing VFA production. The inclusion of these isolates enhanced *in vitro* rumen fermentation and modulated the microbial community, implying beneficial effects on animal feeding applications, with each strain exhibiting unique benefits.

## 1 Introduction

The use of probiotics or direct-fed microbials (DFM) as feed additives has shown potential to promote short-chain fatty acid synthesis by rumen microbiota and enhance livestock production efficiency (1–3), as well as a promising methane mitigation strategy by shifting fermentation pathways away from methanogens (4, 5). Rumen-originating isolates have been recognized as probiotic DFM candidates with beneficial functional properties in the host digestive tract (6). In particular, lactic acid bacteria (LAB), such as *Streptococcus* and *Pediococcus* isolated from the rumen, exhibit positive effects on animal health (7, 8). However, rumen microbiota responses and the extent of the positive effects of rumen-origin DFM supplementation have not been consistent (9). Various factors, including microbial taxa and isolation source, host status, and dietary composition (10) may influence the fermentation effects of the respective isolates and their inclusion levels (11–13). A single species may exert opposite effects in the rumen environment compared to pure culture conditions. For example, LAB may contribute to pH regulation by promoting the fermentation of lactate to propionate by lactate-utilizing bacteria in the rumen (14).

Various nutritional and feeding-related problems arise in goat production. For example, heavy metal loads in feed and imbalances in metabolic minerals within the animals (15), and gastrointestinal disorders caused by overfeeding of concentrated feed to ensure high productivity (16). Regarding the latter, the introduction of DFM has been shown to be a practical solution (17). Moreover, the rumen bacterial community of goats is rich and diverse, making it a promising source of probiotic candidates, and supplementation may beneficially regulate the rumen microbiome. In particular, by introducing potent isolates to goats can improve their carbohydrate degradation ability and utilize a variety of low-quality feed resources (18).

We hypothesized that goat rumen-derived isolates, particularly LAB, would modulate rumen fermentation in distinct ways. The objective of the present study was to characterize isolates belonging to three taxa of LAB obtained from the rumen of adult goats, and to evaluate their effects on *in vitro* rumen fermentation. Microbial community analyses combined with PICRUSt2-based predicted metabolic pathway mapping using the Kyoto Encyclopedia of Genes and Genomes (KEGG) database were used to identify the distinct regulatory patterns associated with different isolates.

## 2 Materials and methods

### 2.1 Bacteria isolation from goat rumen

Animal handling procedures followed Shinshu University guidelines, and approval was obtained for routine husbandry and rumen fluid sampling (No. 020089).

Rumen fluid samples were obtained from four healthy, adult, Japanese native-breed (Shiba) goats at Shinshu University shown in Supplementary Table 4 for properties of the goats. Donor animals were fed oat hay and alfalfa hay cubes twice daily, at 09:00 and 15:00. Rumen fluid was collected using a pump and oral stomach tube the morning feeding. Approximately 50 mL of the collected rumen fluid was discarded to avoid saliva contamination. The collected ruminal fluid was flushed with CO_2_ and processed within 2 h of sampling. Three commercial media formulations were used to isolate and grow rumen bacteria. Brain Heart Infusion broth (BHI; Difco Laboratories, Detroit, MI, USA) was used as a nutrient-rich general medium to recover a broad range of anaerobic cultures (19–21). The de Man, Rogosa, and Sharpe agar (MRS; Oxoid, Basingstoke, UK) was used for lactic acid-producing bacteria (22). Gifu Anaerobic Medium (GAM; Nissui Pharmaceutical Co., Ltd., Tokyo, Japan) has been used for the culture and isolation of anaerobic bacteria in many studies and a variety of microorganisms have been successfully isolated (23, 24).

Each medium was prepared using 40% autoclaved clarified rumen fluid and 15 g agar to solidify the medium. Autoclaved clarified rumen fluid was used as a rumen-derived nutrient supplement to provide nutrients and growth-supporting components and to create a more rumen-relevant nutritional background for bacterial isolation (25). Clarified ruminal fluid was prepared according to previously-reported methods (26–28) Clarified rumen fluid was prepared by centrifugation, followed by autoclaving, before addition to the isolation media. Bacteria were isolated using standard dilution plating on prepared agar plates, followed by 48 h of incubation at 39 °C using the AnaeroPack system (AnaeroPack-Anaero, Mitsubishi Gas Chemical Co., Inc., Tokyo, Japan) (29). Streak plate purification was conducted using the appropriate medium.

Pure cultures were obtained after three rounds of purification. Fifteen colonies were isolated from each of the three types of culture media. The candidate isolates were screened using a combination of qualitative and quantitative analyses. Qualitative screening included colony morphology, size, source medium, and growth characteristics during purification. The isolates were subjected to a preliminary 16S rDNA gene sequence identification. After 24 h of cultivation, culture samples were centrifuged, and the supernatants were collected for quantitative measurements, including final culture pH (pH meter, LAQUAtwin-pH-22, HORIBA, Kyoto, Japan). Organic acid concentrations were measured using high-performance liquid chromatography as described in 2.5. Based on the combined taxonomic diversity and preliminary metabolic characteristics, four representative isolates were selected from the identified genera for further analysis. Although isolates 7 and 14 both belonged to the genus *Streptococcus*, they were retained because they exhibited different preliminary acidification and metabolic profiles. Candidate isolates with different taxonomic identities and metabolic characteristics were selected.

### 2.2 Characterization of isolate metabolic products

The organic acid content of each isolated strain was measured. Single colonies of each isolate were picked from a Petri dish, inoculated into 10 mL of corresponding broth in a test tube, and incubated at 39 °C. The initial pH of each corresponding broth before inoculation and the pH of culture samples was determined using a pH meter. Organic acids were measured via high-performance liquid chromatography using an LC-2000 system as described 2.5. A commercial kit (F-Kit Ammonia, Roche Diagnostics, Tokyo) was used for ammonia nitrogen (NH_3_-N) determination following to the instructions.

### 2.3 Molecular identification of isolates

DNA was extracted for molecular strain identification using a QIAamp DNA Stool Mini Kit (Qiagen, Hilden, Germany) following the manufacturer’s instructions. Extracted DNA was stored at −20 °C until analysis. The 16S rRNA gene sequence from each isolate was amplified, performed sequencing using an Applied Biosystems 3730xl DNA analyzer (Applied Biosystems Inc., CA, USA) using a “MicroSEQ” package. For the first PCR were 8F (5′-AGAGTTTGATCMTGGCTCAG-3′) and 1492R (5′-GGTTACCTTGTTACGACTT-3′), which were used to amplify wider coverage of the 16S rRNA gene sequence. A T100 (Bio-Rad Inc.) thermocycler was used to generate the amplicons. Thermocycler conditions for amplification were as follows: initial denaturation at 95 °C for 10 min, and 30 cycles of 95 °C for 30 s, 60 °C for 30 s, and 72 °C for 45 s for the PCR to make a nearly full length amplicons. Secondly, 515F (5′-GTGCCAGCMGCCGCGGTAA-3′) and 806R (5′-GGACTACHVHHHTWTCTAAT-3′) were used for the cycle sequencing to obtain a contig sequence nearly 1,300 bp reading frame with a 291 bp (i.e., position 515-806) overlap. Sequenced data were compared with those of closely related species using the BLAST platform retrieved from the NCBI nucleotide database. The sequencing data were deposited in the DNA Data Bank of Japan Sequence Read Archive under BioProject accession number PRJDB42109. A phylogenetic tree was constructed using MEGA software and neighbor-joining and maximum-likelihood methods (30).

### 2.4 *In vitro* experiments

Four isolates belonging to different genera were selected to evaluate their effects on ruminal fermentation using a 24 h *in vitro* batch culture system, which can rapidly assess the direct effects of isolate on rumen fermentation and microbial community, and is considered a cost-effective model (31). The concentration of each isolate was measured by plate counting and reported as colony-forming units per milliliter (CFU/mL) (22). A two-factor experiment was conducted. The factors included the four isolates and three inoculation doses (10^4^, 10^6^, and 10^8^ CFU per bottle), with no isolate added as a control. Each treatment was performed in triplicate. Rumen fluid for *in vitro* fermentation assays was collected from the same three healthy adult Japanese Shiba goats described above, 2 h after morning feeding. Rumen fluid from three donor goats was pooled before incubation and infused with CO_2_ to maintain an anaerobic environment. The collected rumen fluid was mixed with pre-warmed McDougall’s buffer (pH 7.5) (32) at a ratio of 1:2 (33) and flushed with N_2_ gas to maintain anaerobic conditions. The same buffered rumen fluid was used for all treatments and the control to ensure a consistent baseline condition. A total of 40 mL of buffered rumen fluid was dispensed into each culture bottle containing 0.4 g substrate (crushed and milled; same amount fed to an animal). The inoculum concentration for each isolate was prepared by centrifugation the activated culture, resuspension, and dilution in PBS to obtain inoculum suspension concentrations of 10^4^, 10^6^, and 10^8^ CFU per bottle (33). Control bottles did not contain any additives. After dispensing, bottles were sealed with rubber stoppers, incubated at 39 °C for 24 h, and shaken automatically at 75 rpm.

### 2.5 Sample collection and analyses

#### 2.5.1 *In vitro* gas production and methane yield

Gas production was measured at 6 and 24 h during incubation, followed by analysis of CH_4_ concentration in the gas to obtain the volume of *in vitro* CH_4_ production at 24 h. The proportion of methane in the generated gas was analyzed using a gas chromatography system (Inorga LC Science Co., Ltd., Nara, Japan). Methane gas volume was calculated using a standard gas mixture and SIC 480 II software for ChromatoLogger (System Instruments Co., Ltd., Hachioji, Japan).

#### 2.5.2 Organic acids, pH, and ammonia nitrogen

Organic acid concentrations were measured using high-performance liquid chromatography using an LC-2000 system (JASCO Corporation, Tokyo, Japan), a solid-phase extraction cartridge (Bond Elute Carbon S, 100 mg, 1 mL, Cat. 5610-2079, Agilent Technologies, Lake Forest, CA, USA) comprising activated charcoal, was used to remove pigments and other materials that could interfere with the UV detection of organic acids in the HPLC loading (34, 35). The procedure for the pretreatment implied conditioning the cartridge with a phosphate buffer (10 mM, pH7.5); (2) add 1 mL of the rumen fluid (or the *in vitro* culture) mixed with a phosphate buffer (50 mM, pH7.5) at a 4:1 ratio to make up a sample in 10 mM phosphate, and collect the effluent for the following HPLC analysis; (3) to remove polar molecules of organic acids retained to the filled charcoal, add 0.5 mL perchloric acid (0.1%) and collect the extract in the same tube of the above effluent. The chromatographic conditions aligned with previously described procedures (36) (column, Inertsil ODS-3 (250 mm × 4.6 mm; GL Science Inc.); oven temperature, 40 °C, mobile phase, 10% acetonitrile with 0.02% perchloric acid; flow rate, 1 mL/min; detection wavelength, 210 nm. Operational control, peak detection, and quantification were conducted using the Chroman software (JASCO Corporation, Tokyo, Japan). Based on the combined taxonomic diversity and preliminary metabolic characteristics, four representative isolates were selected from the identified genera for further analysis. Although isolates 7 and 14 both belonged to the genus *Streptococcus*, they were retained because they exhibited different preliminary acidification and metabolic profiles. Candidate isolates with different taxonomic identities and metabolic characteristics were selected. NH_3_-N concentration was determined using the commercial kit described above. Rumen pH was measured using a pH meter.

#### 2.5.3 Dry matter digestibility

At the end of the incubation period (24 h), culture contents were filtered through a previously weighed quantitative ashless filter paper (grade 5A, 110 mm in diameter; ADVANTEC, Toyo Roshi Kaisha, Ltd., Tokyo, Japan) using a vacuum pump and a Buchner funnel. Residues were dried at 65 °C for 48 h and *in vitro* dry matter digestibility (IVDMD) was calculated as follows:


$$\begin{array}{l} \text{IVDMD}\;(\%)\\ =[1-(\begin{array}{l} \left(\mathrm{DM}\;\text{residue sample}-\mathrm{DM}\;\text{residue blank}\right)\\ /\text{initial}\;\mathrm{DM}\;\text{of the substrate} \end{array})]\times 100 \end{array}$$

#### 2.5.4 Bacteria and archaea qPCR, amplicon sequencing

The primer sets Eub338F (ACTCCTACGGGAGGCAG) and Eub522R (ACGTCRTCCMCNCCTTCCTC) were used to quantify total bacteria, whereas qmcrA-F (TTCGGTGGATCDCARAGRGC) and qmcrA-R (GBARGTCGWAWCCGTAGAATCC) were used to quantify archaea. Forty PCR cycles (denaturation at 95 °C for 10 s, annealing at 60 °C for 20 s, and extension at 72 °C for 30 s) were performed. Dissociation and melting curve analyses were performed to confirm the presence of the expected PCR products. Extracted bacterial genomic DNA of replicate cultivations was mixed in equal proportions and subjected to 16S rRNA gene amplicon sequencing (37). The primer set for the first PCR was 515F (5′-GTGCCAGCMGCCGCGGTAA-3′) and 806R (5′-GGACTACHVHHHTWTCTAAT-3′), with the 5′-end of 806R connected to the universal tag sequence for the following PCR. The T100 thermal cycler (Bio-Rad Inc.) and ExTaq (Takara Bio Inc.) were used to generate amplicons. The thermal cycler conditions used for amplification were set as follows: initial denaturation at 95 °C for 10 s, and 25 cycles of 95 °C for 10 s, 57 °C for 30 s, and 72 °C for 30 s for the first PCR to make the amplicons with the universal tag for second PCR primer sets, whereas 10 cycles of 95 °C for 10 s, 57 °C for 30 s, and 72 °C for 30 s for the second PCR for obtaining barcoded amplicons suitable for an Illumina MiSeq platform (Illumina, San Diego, CA, USA). The second amplicons were subjected to paired-end sequencing, and the sequencing data were deposited as the same ID in the depository.

#### 2.5.5 Microbial community

Replicate culture samples from the same isolate × dose condition were manually pooled before sequencing. A total of 15 composite sequencing samples were obtained, corresponding to five treatment groups, including the control, isolate 11, isolate 14, isolate 7, and isolate B groups, with three dose-level composite samples within each treatment group. Because the effects of inoculation dose on *in vitro* fermentation parameters were generally limited, the microbial community analysis was performed according to isolate treatment groups. Microbial community analysis was conducted based on amplicon sequence variants (ASVs). Clean tags were denoised using the DADA2 plug-in for QIIME2 software. Relative abundance and diversity analyses were conducted after total-sum scaling normalization (38). Alpha diversity indices (including Ace, Chao 1, Shannon, Simpson) were used to analyze species diversity within samples. Principal coordinate analysis (PCoA) based on the Bray-Curtis distance algorithm, together with PERMANOVA, was used to evaluate the significance of differences in community structure (39). LEfSe analysis (LDA > 2, *p* < 0.05) was performed to identify discriminatory bacterial taxa at family and genus levels, following previous studies (40–42), the Kruskal–Wallis test was used to identify species showing significant abundance differences among groups, and the Wilcoxon rank-sum test was used to evaluate differences between groups. PICRUSt2 was used to predict potential microbial functional profiles using the Kyoto Encyclopedia of Genes and Genomes (KEGG) database (39).

### 2.6 Statistical analysis

Data from the *in vitro* experiments were analyzed by two-way analysis of variance (ANOVA) using IBM SPSS Statistics (version 26.0, IBM Corp, Armonk, New York, USA). Isolate × dose factorial analysis included only the isolates and dose treatments and excluded the control, using the following model:


Yijk=μ+αi+βj+(αβ)ij+εijk

where *Y_ijk_* is the observed value, *μ* is the overall mean, *α_i_* is the fixed effect of isolates, *β_j_* is the fixed effect of dose, (*αβ*)*_ij_* is the interaction effect between isolates and dose, and *ε_ijk_* is the residual error. In microbial community analyses, *β_j_* was dealt with random effects. The *p*-values for isolate, dose, and isolate × dose interaction were obtained from this two-way ANOVA. Simple effect comparisons were performed using Sidak adjustment when needed. Comparisons between each treatment and the control were conducted separately using Dunnett’s adjustment, and showed as starred superscripts. Differences were considered statistically significant at *p* < 0.05. Polynomial (linear and quadratic) contrasts were used to examine dose responsiveness. Alpha diversity indices were calculated in R using the vegan package, and were compared among treatment groups using Kruskal–Wallis test. All microbial data analyses and visualizations were performed using R software (version 4.4.2).

## 3 Results

### 3.1 Isolate identification

**Figure 1:**
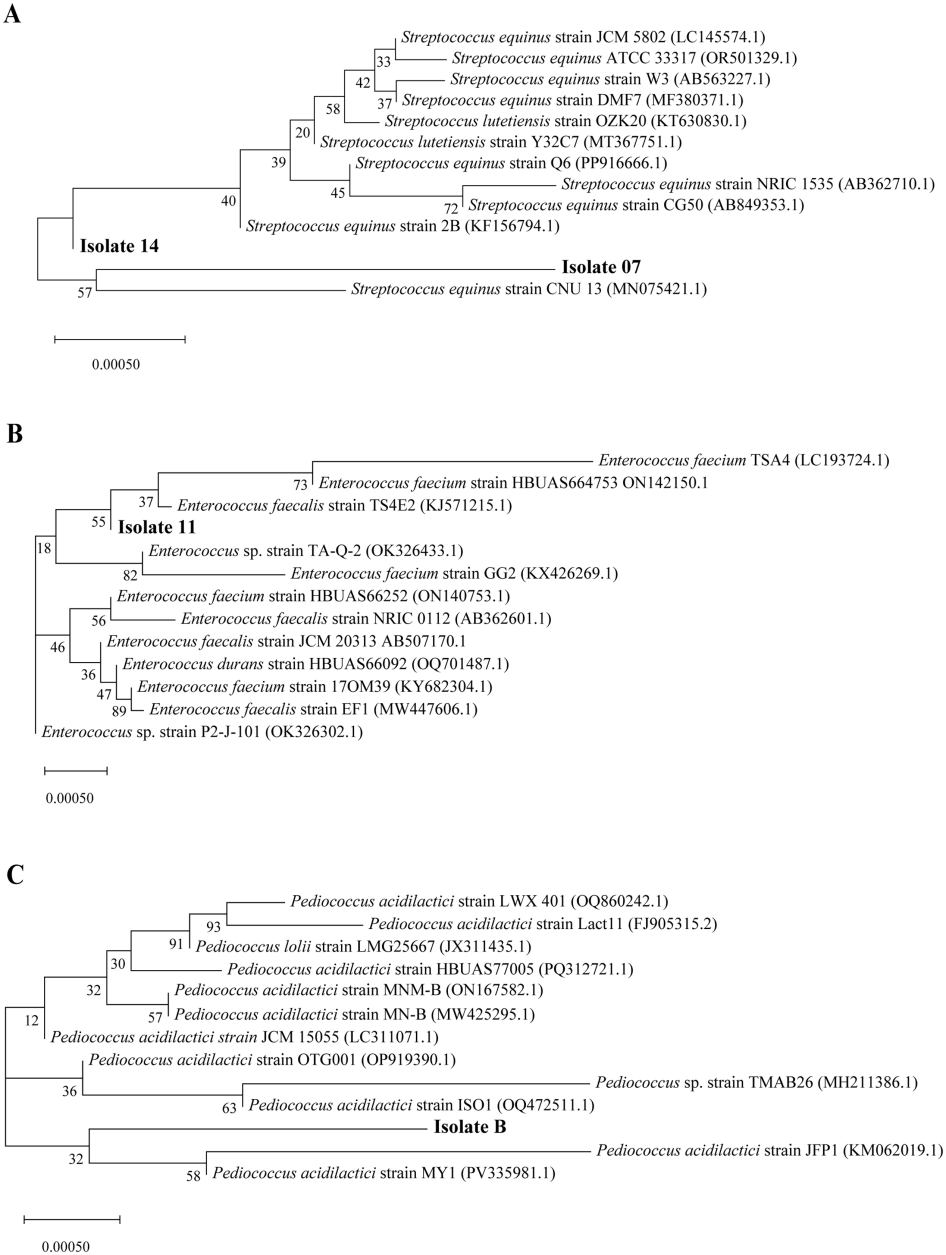
The maximum-likelihood phylogenetic tree of four isolates based on partial 16S rRNA gene sequences of *Streptococcus* spp. (isolate 7 and 14, **A**), *Enterococcus* spp. (isolate 11, **B**), and *Pediococcus* spp. (isolate **B, C**). Bootstrap values were shown at branch points.

Four representative isolates were selected and identified by 16S rRNA gene sequence analysis. The closest BLAST matches showed high sequence similarity to *Enterococcus faecium* strain GG2 for isolate 11 (99.93%)*, Streptococcus equinus* strain 2 B for isolate 14 (99.86%), *Streptococcus equinus* strain CNU 13 for isolate 7 (99.86%), and *Pediococcus acidilactici* strain JFP1 for isolate B (99.73%; Supplementary Table 1). Combined with phylogenetic analysis (Figure 1), these results allowed preliminary assignment of isolate 11 to the genus *Enterococcus,* isolates 14 and 7 to the genus *Streptococcus,* and isolate B to the genus *Pediococcus.*

### 3.2 Metabolite analysis

The four isolates displayed distinct preliminary metabolite profiles after 24 h of incubation (Table 1). Isolate B exhibited the numerically lowest final pH and the numerically highest NH_3_-N concentration. Isolate 11 produced the relatively higher lactate and isolate 14 produced the least. Isolate 7 maintained relatively high pH and butyric acid concentrations.

**Table 1:** Metabolic characteristics of isolates after 24 h incubation.

Item	Bacteria 11	Bacteria 14	Bacteria 7	Bacteria B
Final pH	5.52	5.34	7.00	4.06
Lactic acid (mg/L)	92.4	17.1	24.1	44.5
Acetic acid (mg/L)	7.82	0.00	9.70	0.00
Butyric acid (mg/L)	0.00	0.70	3.93	1.23
NH_3_-N (mg/100 mL)	1.20	1.08	1.36	4.05
Culture medium	GAM	GAM	BHI	MRS
Initial medium pH	7.48	7.48	7.55	6.52

### 3.3 *In vitro* fermentation parameters

Significant isolate × dose interactions were detected for pH, total gas production, methane production, and digestibility (Table 2). Compared with control fermentations, isolates 11 and 14 increased total gas production at all inoculation doses. Isolate 14 yielded significantly higher total gas production than the other isolates at 10^4^ CFU. At 10^6^ and 10^8^ CFU, isolate 7 produced significantly less total gas than the other isolates, with its lowest value at 10^6^ CFU. Significant interaction between isolate and dose was observed for CH_4_ production. Simple effect analyses showed that at 10^6^ CFU, isolate 7 produced significantly less methane than isolates 11 and 14. While isolate 7 produced a higher proportion of methane than the other isolates and the control. With isolate B, the methane yield increased linearly with increasing dose Supplementary Table 2, and the methane percentage tended to be lower at 10^4^ and 10^6^ CFU compared to the control. Isolate 14 exhibited a significant quadratic decrease in methane percentage. No significant difference in digestibility was observed compared to the control.

**Table 2:** Effects of isolates and inoculation dose on in vitro rumen pH, gas and methane production and digestibility.

Item	Control	Bacteria 11			Bacteria 14			Bacteria 7			Bacteria B			SEM	*p* value		
		10^4^	10^6^	10^8^	10^4^	10^6^	10^8^	10^4^	10^6^	10^8^	10^4^	10^6^	10^8^		Dose	Bacteria	Interaction
pH	7.00	6.93	6.94	6.93	6.96	6.87^**^	6.91	6.93	6.99	6.95	6.90^*^	6.87^***^	6.84^***^	0.024	0.190	<0.001	0.005
24 h gas production (mL/g substrate)	137.6	147.8^**^	151.7^***^	148.0^**^	159.5^***^	151.6^***^	150.7^***^	144.6	132.7	137.9	143.6	148.6^**^	153.7^***^	2.567	0.226	<0.001	<0.001
Methane %	12.54	13.05	12.75	12.80	13.05	12.71	13.25^*^	13.21	12.83	13.46^**^	12.07	12.36	12.59	0.235	0.030	<0.001	0.236
Methane (mL/g substrate)	18.57	20.69^*^	20.69^*^	20.31	22.19^***^	20.62^*^	21.38^***^	20.49^*^	18.38	19.99	18.62	19.68	20.67^*^	0.593	0.070	<0.001	0.015
Digestibility%	45.97	45.37	46.67	42.18	44.31	43.47	44.02	42.32	47.07	47.62	45.33	43.72	42.58	1.769	0.385	0.249	0.016

*p < 0.05, **p < 0.01, ***p < 0.001 according to Dunnett’s test against a control (CON). *p*-values for isolate, dose, and isolate × dose interaction were obtained from two-way ANOVA (without control).

All isolates showed increased total VFA concentrations, and isolate B yielded a higher total VFA concentration than the other isolates (Table 3). Total VFA concentration was significantly higher than that of the control for isolate 14 at 10^8^ CFU and for isolate B at 10^6^ CFU and 10^8^ CFU. Regarding the VFA production pattern in molar proportion, only the isolate effect was significant for acetate proportion (AA%), propionate proportion (PA%), butyrate proportion (BA%), and acetate-to-propionate ratio (A/P). In cultures with isolate 14, AA% decreased with increasing dose. Isolates B and 14 showed significantly higher PA% than isolates 11 and 7. Isolates 11, 14, and 7 yielded significant increases in BA% compared with the control, whereas isolate B exhibited the lowest BA% among the isolates and the control. All inoculated isolates showed lower A/P ratios than the control. None of the treatments resulted in significant differences in bacterial copy number compared to the control (Table 4). Fermentations inoculated with isolates 7 and B exhibited significantly lower archaeal abundances than the control fermentation or those inoculated with isolates 11 or 14 (Table 5).

**Table 3:** Effects of isolates and inoculation dose on rumen in vitro VFA profiles.

Item	Control	Bacteria 11			Bacteria 14			Bacteria 7			Bacteria B			SEM	*p* value		
		10^4^	10^6^	10^8^	10^4^	10^6^	10^8^	10^4^	10^6^	10^8^	10^4^	10^6^	10^8^		Dose	Bacteria	Interaction
AA%	60.27	57.72^***^	58.64^**^	58.29^***^	57.52^***^	57.19^***^	56.40^***^	58.94^*^	58.52^**^	58.63^**^	58.32^**^	58.31^***^	57.22^***^	0.436	0.133	<0.001	0.272
PA%	30.15	31.79^*^	30.84	31.30	32.13^**^	32.51^***^	33.14^***^	30.97	31.41	31.26	32.50^***^	32.35^***^	33.50^***^	0.473	0.212	<0.001	0.428
BA%	9.58	10.49^***^	10.52^***^	10.41^***^	10.36^***^	10.30^***^	10.46^***^	10.09^***^	10.07^***^	10.11^***^	9.18^**^	9.34	9.28^*^	0.095	0.815	<0.001	0.614
Total VFA (mmol/L)	48.49	53.56	53.51	53.67	52.84	51.03	56.10^*^	52.32	50.67	54.11	54.01	55.75^*^	57.90^***^	2.093	0.130	0.181	0.847
A/P	2.000	1.816^*^	1.902	1.863	1.790^**^	1.759^***^	1.707^***^	1.903	1.863	1.876	1.794^**^	1.802^*^	1.711^*^	0.039	0.192	<0.001	0.324

*p < 0.05, **p < 0.01, ***p < 0.001 according to Dunnett’s test against a control (CON). AA%, acetic acid percentage; PA%, propionic acid percentage; BA%, butyric acid percentage; A/P, acetic acid-to-propionic acid ratio; SEM, standard error of the mean.

**Table 4:** Effects of isolates and inoculation dose on in vitro rumen fermentation bacterial abundances and ammonia nitrogen.

Item	Control	Bacteria 11			Bacteria 14			Bacteria 7			Bacteria B			SEM		*p* value	
		10^4^	10^6^	10^8^	10^4^	10^6^	10^8^	10^4^	10^6^	10^8^	10^4^	10^6^	10^8^		Dose	Bacteria	Interaction
Bacteria (10^7^ copies/mL)	205	205	168	211	221	233	256	225	225	255	247	210	229	21.25	0.049	0.010	0.496
Archaea (10^7^ copies/mL)	8.39	8.23	7.05	9.01	9.87	9.63	7.65	2.53^***^	2.84^***^	2.92^***^	2.82^***^	2.28^***^	2.09^***^	0.996	0.726	<0.001	0.413
NH_3_-N (mg/100 mL)	29.77	29.60	29.85	29.81	29.81	29.61	29.96	29.60	29.45	29.64	29.75	29.60	29.73	0.238	0.527	0.532	0.916

*p < 0.05, **p < 0.01, ***p < 0.001 according to Dunnett’s test against a control (CON).

**Table 5:** Effect of isolates on microbial community on alpha diversity index.

Index	Control	Isolate 11	Isolate 14	Isolate 7	Isolate B	*p* value
Chao1	989.3	1,032.6	998.8	856.9	715.7	0.068
ACE	988.4	1,032.7	998.8	856.8	715.8	0.068
Shannon	5.541	5.606	5.597	5.569	5.477	0.329
Simpson	0.990	0.991	0.991	0.991	0.991	0.095

### 3.4 Microbial community

**Figure 2:**
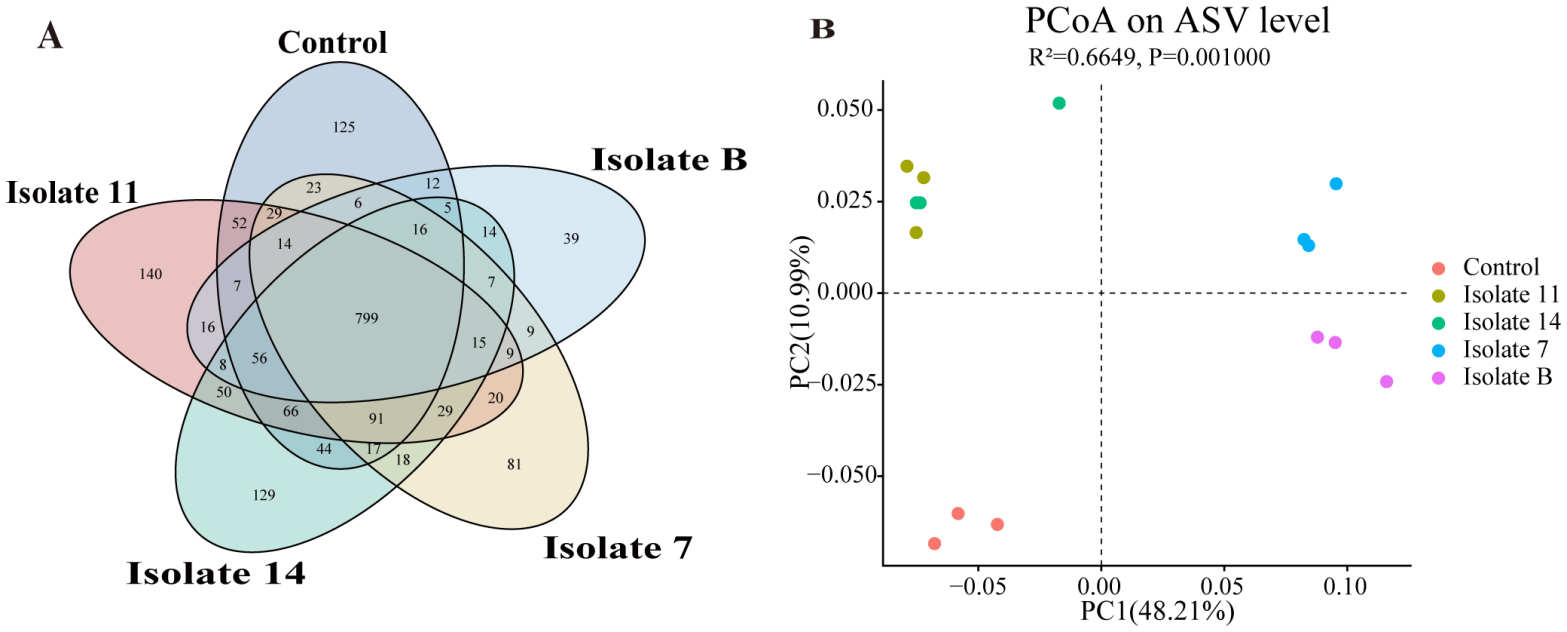
Transformed information about bacterial community structure in in vitro cultures. A Venn diagram showing the unique and shared ASVs of each group and the combinations **(A)**. Principal coordinate analysis (PCoA) of samples at the operational taxonomic unit level using the weighted Unifrac distance metric, with sample attributions indicated in the key **(B)**.

After DADA2 processing, including denoising, merging, and chimera removal, 1,204,604 non-chimeric reads were obtained from 15 samples, 1946 Amplicon sequencing variants (ASV) features were retained across all samples. The Venn diagram in Figure 2A reveals 799 features shared among the five groups. The rarefaction curve gradually approached a plateau with increasing number of reads, indicating that the sequencing coverage was sufficient to capture most of the bacterial diversity in the samples (Supplementary Figure 1). No significant differences in alpha diversity indices were observed among the groups (Table 3). Bray-Curtis-based PCoA showed clear separation between the isolates and the control (Figure 2B), and significant differences in microbial community structures were observed. Pairwise comparisons between the isolates and the control showed a trend toward significance, whereas isolates 11 and 14 showed no significant structural differences (Supplementary Table 3).

**Figure 3:**
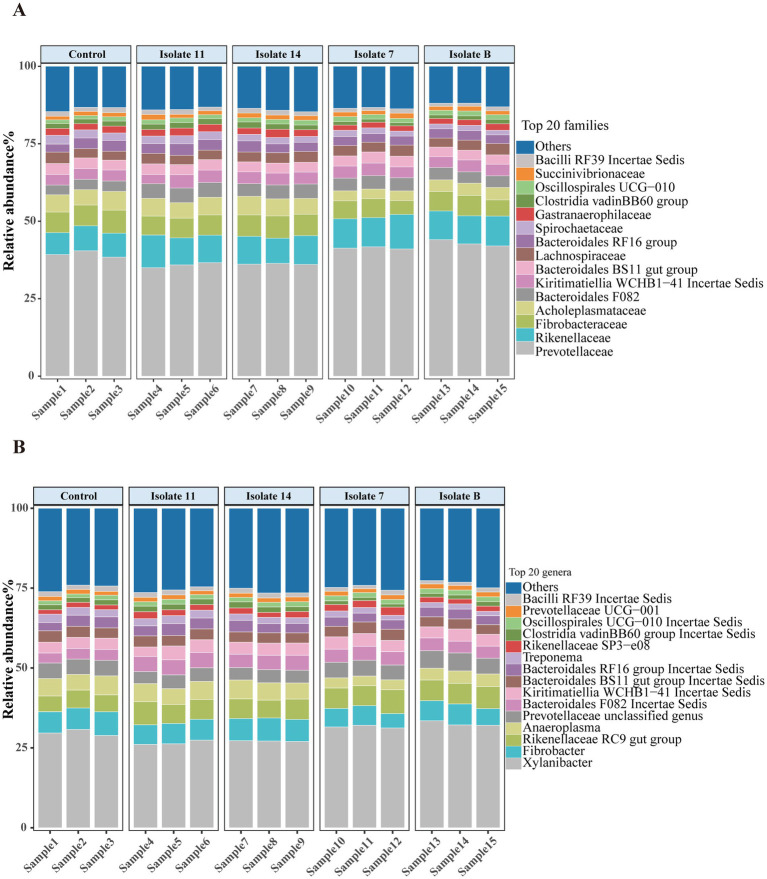
Bacterial composition of bacterial communities in phyla **(A)** and genus **(B)** whose relative abundance belonged to the top 20 in the microbial community.

At the taxonomic level, all groups showed the same overall taxonomic composition, with differences among treatments mainly reflected in shifts in relative abundance. In total, 22 phyla, 117 families, 217 genera, and 373 species were detected among the rumen microbiotas. At the phylum level, Bacteroidetes, Bacillota, Fibrobacterota, Verrucomicrobiota, and Pseudomonadota were the dominant bacteria, with relative abundances of >90% (Supplementary Figure 2). Bacteroidetes were more abundant in fermentations containing isolates 7 and B than in the control or those containing isolates 11 and 14. Bacillota showed the opposite pattern, being lower in isolates 7 and B. Spirochaetota was highest in the control and isolate 11 groups (Table 6). At the family level (Figure 3A), the top 20 families accounted for more than 85% of total relative abundance. The remaining families were combined with the others. As shown in Table 6, Prevotellaceae abundance differed significantly among the groups and was higher in fermentations containing isolates 7 and B than in those containing the other groups. Fibrobacteraceae differed among groups, with the lowest abundance observed in isolate 7. Acholeplasmataceae were markedly lower in fermentations containing isolates 7 and B than in the control and isolates containing 11 and 14. Synergistaceae was enriched in fermentations containing isolates 7 and B compared with the control and isolate 11. At the genus level (Figure 3B), *Xylanibacter* was the most abundant genus in all groups, followed by *Fibrobacter* and Rikenellaceae_RC9_gut groups. As shown in Table 6, compared with the control, isolates B and 7 significantly increased the relative abundance of *Xylanibacter* but significantly decreased the relative abundances of *Anaeroplasma* and Rhodospirillales_Incertae_Sedis. In addition, isolate B significantly decreased the relative abundance of *Treponema.* Isolates 11 and 14 exhibited similar patterns, both significantly increasing the relative abundance of Bacteroidales_F082 and Prevotellaceae_UCG-003, while decreasing the relative abundance of Prevotellaceae_ unclassified_genus. At the species level, isolate B significantly increased the relative abundance of *Prevotella ruminicola* compared to that in the control. All treatments also significantly increased the relative abundance of *Anaeroplasma abactoclasticum* relative to that in the control.

**Table 6:** Effects of isolates on relative abundance.

Item	Control	11	14	7	B	*p* value	SEM
Phylum level							
Bacteroidota	59.08^b^	59.05^b^	58.02^b^	64.16^a^	64.14^a^	<0.001	0.840
Bacillota	18.88^a^	19.10^a^	19.56^a^	15.86^b^	16.23^b^	0.002	0.773
Fibrobacterota	6.930^ab^	6.327^ab^	7.005^a^	5.483^b^	6.021^ab^	0.034	0.450
Cyanobacteriota	2.140^ab^	2.309^a^	2.278^a^	1.728^b^	1.880^ab^	0.020	0.164
Spirochaetota	3.101^a^	3.031^a^	2.543^b^	2.499^bc^	2.172^c^	<0.001	0.107
Synergistota	0.196^b^	0.223^b^	0.336^ab^	0.512^a^	0.431^a^	<0.001	0.056
Pseudomonadota	2.779^ab^	2.787^ab^	3.165^a^	2.789^ab^	2.294^b^	<0.001	0.180
Family level							
Prevotellaceae	39.39^b^	35.86^c^	36.21^c^	41.37^ab^	42.93^a^	<0.001	0.632
Rikenellaceae	7.600^b^	9.369^ab^	8.778^ab^	9.995^a^	9.294^ab^	0.025	0.599
Bacteroidales_F082	3.279^d^	4.748^a^	4.417^ab^	4.226^bc^	3.813^c^	<0.001	0.134
Bacteroidales_BS11_gut_group	3.411	3.379	3.209	3.447	3.104	0.026	0.099
Fibrobacteraceae	6.930^ab^	6.327^ab^	7.005^a^	5.483^b^	6.021^ab^	0.034	0.450
Gastranaerophilaceae	2.130^ab^	2.307^a^	2.259^a^	1.714^b^	1.869^ab^	0.019	0.163
Acholeplasmataceae	5.520^a^	5.466^a^	5.542^a^	3.150^b^	3.917^b^	<0.001	0.304
Synergistaceae	0.196^b^	0.223^b^	0.336^ab^	0.512^a^	0.431^a^	<0.001	0.056
Spirochaetaceae	2.625^a^	2.546^a^	2.040^b^	1.85^bc^	1.575^c^	<0.001	0.125
Clostridia_vadinBB60_group_Incertae_Sedis	1.571^ab^	1.766^a^	1.725^a^	1.057^c^	1.296^bc^	<0.001	0.103
Erysipelatoclostridiaceae	1.237^a^	1.395^a^	1.414^a^	0.809^b^	1.082^ab^	0.003	0.123
Rhodospirillales_Incertae_Sedis	1.391^a^	1.278^a^	1.316^a^	0.951^b^	0.816^b^	<0.001	0.096
Bacteroidales_unclassified_family	0.905^a^	0.857^a^	0.854^a^	0.672^b^	0.652^b^	<0.001	0.045
Clostridia_UCG014_Incertae_Sedis	0.749^ab^	0.803^a^	0.881^a^	0.791^ab^	0.593^b^	0.011	0.062
Bacteroidales_p251o5	0.442^c^	0.52^bc^	0.537^b^	0.716^a^	0.542^b^	<0.001	0.029
Genus level							
*Xylanibacter*	29.78^c^	26.59^c^	27.13^b^	31.61^a^	32.57^a^	<0.001	0.547
*Fibrobacter*	6.926^ab^	6.324^ab^	7.003^a^	5.482^b^	6.019^ab^	0.034	0.449
Rikenellaceae RC9 gut group	5.259^a^	6.490^ab^	6.034^ab^	6.720^a^	6.608^ab^	0.031	0.415
*Anaeroplasma*	5.439^a^	5.400^a^	5.462^a^	3.075^b^	3.844^b^	<0.001	0.293
Prevotellaceae unclassified genus	4.852^a^	4.122^b^	4.016^b^	4.815^a^	5.344^a^	<0.001	0.191
Bacteroidales F082 Incertae Sedis	3.279^d^	4.748^a^	4.417^ab^	4.226^bc^	3.813^c^	<0.001	0.134
Bacteroidales BS11 gut group Incertae Sedis	3.411^ab^	3.379^ab^	3.209^ab^	3.447^a^	3.104^b^	0.026	0.099
*Treponema*	2.447^a^	2.412^a^	1.936^b^	1.714^bc^	1.470^c^	<0.001	0.132
Rikenellaceae SP3-e08	1.471^b^	1.910^ab^	1.770^ab^	2.253^a^	1.613^b^	0.004	0.154
Clostridia vadinBB60 group Incertae Sedis	1.571^ab^	1.766^a^	1.725^a^	1.057^c^	1.296^bc^	<0.001	0.103
Prevotellaceae UCG-003	1.062^b^	1.452^a^	1.453^a^	1.282^ab^	1.285^ab^	0.025	0.108
Rhodospirillales Incertae Sedis	1.391^a^	1.278^a^	1.316^a^	0.950^b^	0.816^b^	<0.001	0.096
Species level							
*Prevotella ruminicola*	0.935^b^	1.035^ab^	1.040^ab^	1.058^ab^	1.116^a^	0.019	0.042
*Anaeroplasma abactoclasticum*	0.574^b^	0.640^a^	0.559^a^	0.345^a^	0.480^a^	<0.001	0.041

Within the same row, values with different superscript letters (**a**–**d**) differ significantly among treatments (*p* < 0.05).

**Figure 4:**
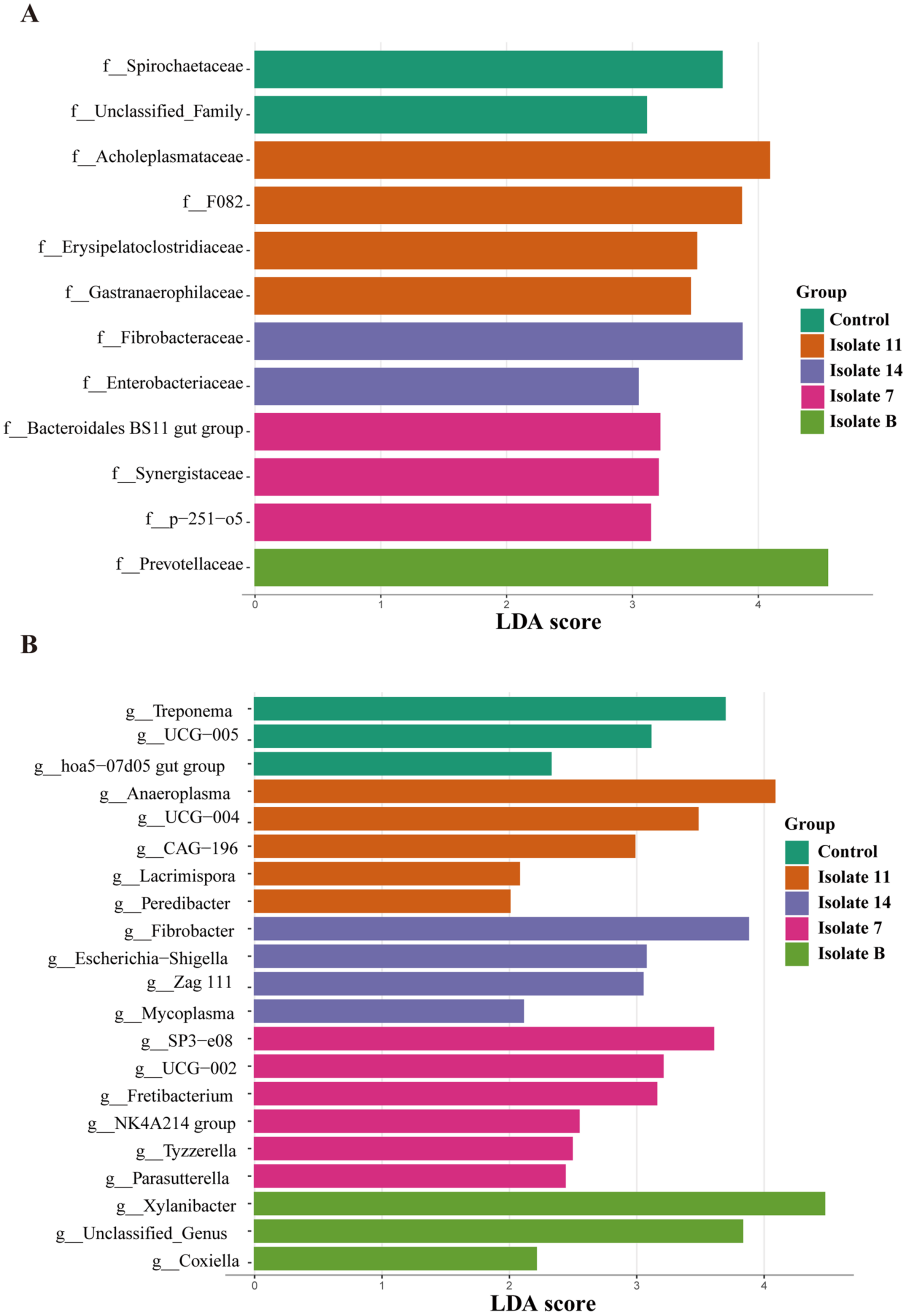
The signature taxa were defined by LEfSe. Indicator bacteria with LDA scores in bacterial communities at family level **(A)** and at genus level **(B)** associated with each group.

**Figure 5:**
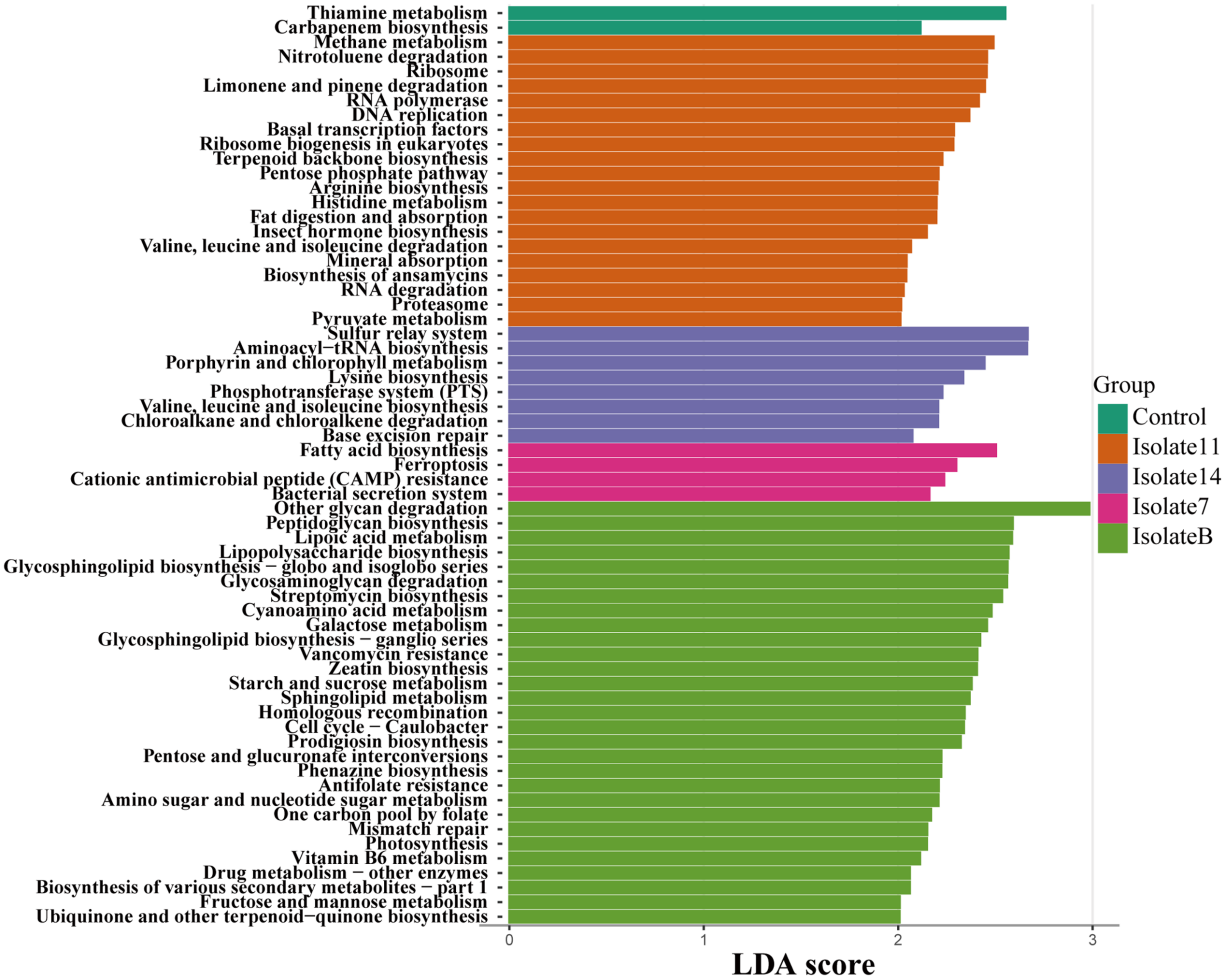
The signature biomarkers were defined by LEfSe. Histogram of the linear discriminant analysis (LDA) scores for differentially abundant features among groups.

LEfSe analysis revealed differential biomarkers (LDA score >2) among the five groups (Figure 4). At the family level (Figure 4A), Acholeplasmataceae, Erysipelatoclostridiaceae, Gastranaerophilaceae, and Bacterovoracaceae were enriched with inclusion of isolate 11, whereas Fibrobacteraceae, Enterobacteriaceae, and Mycoplasmataceae were enriched with isolate 14. The Bacteroidales BS11 gut group, Synergistaceae, Anaerovoracaceae, and Sutterellaceae were identified as the main discriminatory families for isolate 7. Prevotellaceae and Coxiellaceae were identified as biomarkers for the group incorporating isolate B. At the genus level (Figure 4B), isolate 11 fermentations were enriched with *Anaeroplasma*, *Lacrimispora* and *Peredibacter*, whereas isolate 14 fermentations were mainly associated with *Fibrobacter* and *Mycoplasma*. Isolate 7 fermentations were characterized by *Fretibacterium*, *Tyzzerella*, and *Parasutterella*. In fermentations including isolate B, *Xylanibacter* and *Coxiella* were identified as discriminatory genera (Figure 5).

**Figure 6:**
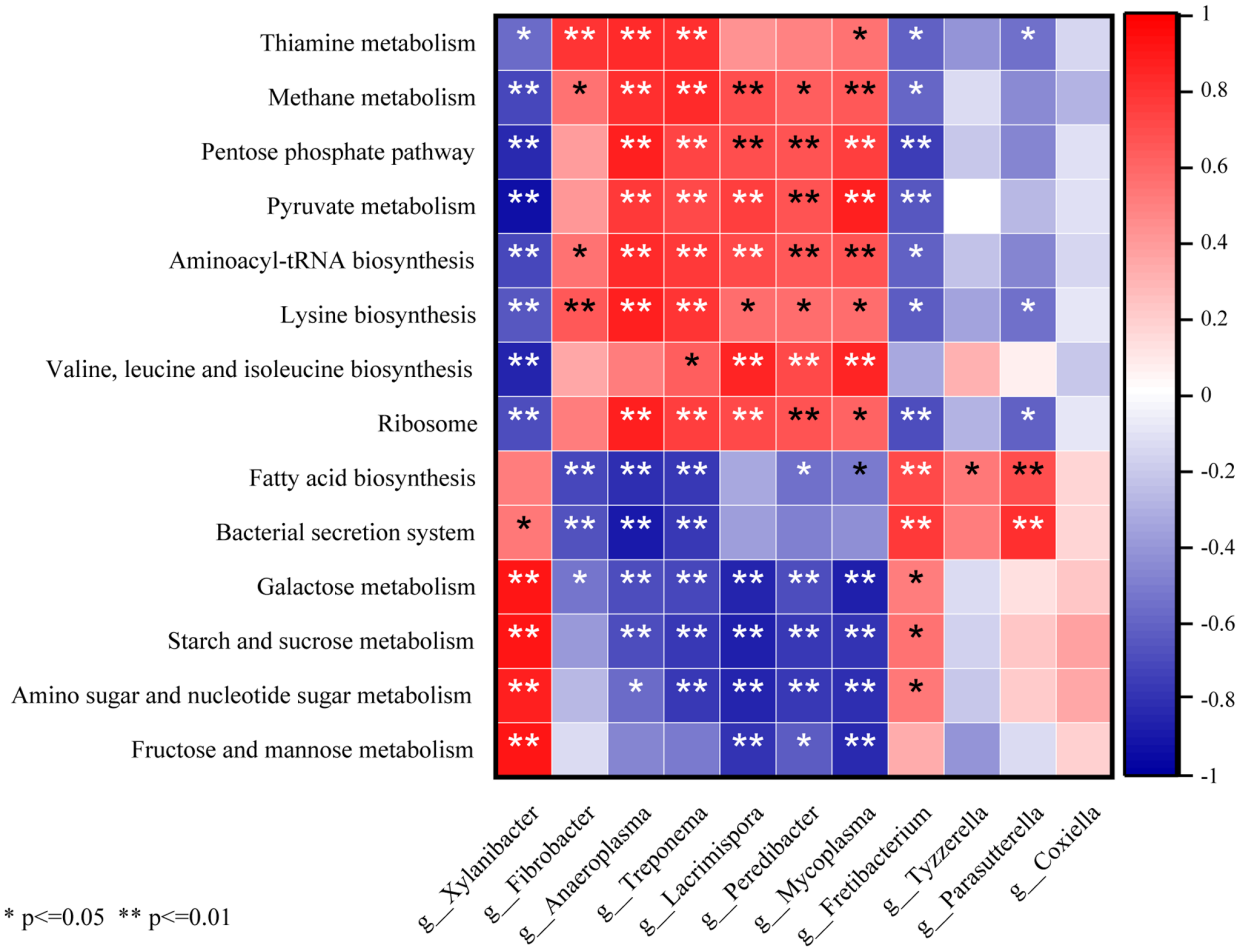
Spearman correlation heatmap between selected discriminatory genera and predicted functional pathways. Matrix cell color represents negative (blue) or positive (red) correlations. * and ** indicate significant differences at *p* < 0.05 and <0.01, respectively.

Forty-four total KEGG level 2 and 307 KEGG level 3 enriched pathways were identified using PICRUSt. At KEGG level 2 (Supplementary Figure 3), the predominant predicted pathways were mainly related to metabolism, with amino acid metabolism being the most enriched pathway (12.69 ± 0.09%), followed by carbohydrate metabolism (11.35 ± 0.05%). The predicted KEGG level 3 pathways were further analyzed using LefSe (Figure 6). The predicted functional potentials further support the conclusion that different isolates are associated with distinct functional shifts. For isolate 11, significant biomarkers of methane and pyruvate metabolism pathways were identified. Isolate 14 was mainly associated with pathways related to aminoayl-tRNA biosynthesis and phosphotransferase systems. Isolate 7 inclusion was characterized by fewer enriched pathways, mainly involving fatty acid biosynthesis and ferroptosis. Isolate B inclusion enriched in multiple pathways related to carbohydrate metabolism, including galactose, starch, and sucrose; amino and nucleotide sugars; and fructose and mannose metabolism.

Correlation analysis between selected discriminatory genera and predicted functional pathways (Figure 6) indicated coordinated relationships between microbial community shifts and inferred functional profiles. Several carbohydrate-related pathways, including galactose metabolism, starch and sucrose metabolism, amino sugar and nucleotide sugar metabolism, and fructose and mannose metabolism, correlated strongly and positively with *Xylanibacter* and *Fretibacterium*. *Fibrobacter* displayed significant positive correlation with thiamine metabolism, aminoacyl-tRNA biosynthesis, and lysine biosynthesis pathways. *Anaeroplasma*, *Treponema*, *Lacrimispora*, *Peredibacter*, and *Mycoplasma* displayed strong positive correlations with methane metabolism, the pentose phosphate pathway, pyruvate metabolism, and ribosomes.

## 4 Discussion

### 4.1 Effect of isolates on *in vitro* rumen fermentation parameters

Supplementation with the isolate enhanced fermentation by increasing the production of total gas and VFA. Since acetate and butyrate formation is well associated with CO₂ production, the decreased proportions of acetate and butyrate may have been linked to reduced CO₂ production, which could partly explain the higher methane percentages and total methane yield after supplementation with the isolates. The marked change in positive methane production was unexpected because bacterial supplementation often reduces rumen methane emissions (43). The kinetics are usually accompanied by a shift in hydrogen utilization toward propionate formation, which was found in all isolates compared to the control. We assumed that this observation was due to bacteria that produced propionates without introducing free H_2_. The two *Streptococcus* isolates (14 and 7) induced fermentation differently, which could not be explained by taxonomic classification but could also indicate strain-specific effects. The probiotic potential of different bacterial strains varies, even within the same species (44).

The isolates substantially affected rumen fermentation parameters *in vitro*, and these effects differed among the isolates. The isolates were expected to act in different or synergistic ways to help fibrolytic microbial consortia promote enzymatic hydrolysis of feed carbohydrates and increase organic acid production (45). isolate supplementation did not markedly affect digestibility, which may be partly related to the low starch availability. As this *in vitro* incubation contained 100% forage as a substrate, the effects of bacterial supplementation on *in vitro* digestibility may vary, particularly with dietary substrate composition (46). For example, digestibility was not markedly affected in a 60% high-forage diet supplemented with bacteria (47). On the other hand, community intervention with the isolates may have simultaneously led to a decrease in gas production and an increase in metabolite VFA production, due to metabolizing different types of substrates were to similar degrees over the same period. Ruminal microorganisms in different taxa can use different types of hexoses (fructose, galactose, and mannose) and pentoses as fermentable substrates to produce various organic acids, such as VFA, lactate, and succinate (48).

### 4.2 Effect of supplementing isolates on predicted function and microbial community

A clear pattern of separation in the microbial community structure was evident using PCoA, suggesting that the isolates may drive the ruminal microbial community in different directions. The distinct metabolic contributions of the four isolates may reflect their putative functional diversity in rumen fermentation, as supplementation would primarily mediate metabolic switching among resident microorganisms rather than their population replacement (49).

Although the predicted functional results should be interpreted with caution, as inferred from 16S rRNA gene data using PICRUSt2, changes in KEGG level 2 metabolism highlighted that the potential effects of the isolates were mainly attributable to amino acid and carbohydrate metabolism, which may indicate a greater capacity for the utilization of specific sugar substrates by community members (50). Various levels of -omics approaches have been applied for address practical issues in goat production (51). In this study, an original approach was adopted under same point of view, and isolates capable of controlling rumen fermentation were obtained from goat rumen, and preliminary screening was conducted exploratory by *in vitro* culture. Although it was rather indirectly implication, changes in rumen fermentation pathway characteristics have been shown. For example, the inclusion of isolate B belonging to *Pediococcus* increased both gas production and VFA levels without DMD changes due to compositional changes in the fermentation substrates within the defined fermentation period (24 h). This may be associated with the enrichment of *Prevotella* in the isolate, possibly activated during starch fermentation and carbohydrate metabolism (52, 53). *Xylanibacter* and *Prevotella*, which are closely related to carbohydrate-utilizing lineages with overlapping ecological characteristics, have been linked to the degradation of different hemicelluloses and plant polysaccharides (54), releasing fermentable sugars and intermediate metabolites that can be utilized subsequently by other bacteria inhabiting the rumen. Heatmap analysis supported this interpretation as *Xylanibacter* showed strong positive correlations with the predicted metabolic pathways of various types of sugars. In a preceding study, isolated *Pediococcus* strain showed improved *in vitro* digestibility (55), supporting the possible relevance of this taxa as used in a DFM the rumen-derived isolates improving other members in metabolic pathway-driven manner.

Isolates 11 and 14 also had the potential to enhance VFA fermentation. Bacteroidales F082 and *Anaeroplasma* were substantially enriched in isolate 11. *Anaeroplasma* is positively correlated with fibrolytic isozyme activity (56). Heatmap analysis indicated that pyruvate metabolism was strongly positively correlated with *Anaeroplasma*, *Fibrobacter,* and *Mycoplasma* via enrichment during fermentation. Enrichment of the ribosomal pathway generally indicates greater protein synthesis potential and more active translational processes (50). Supplementation with isolate 11 increased microbial metabolic activity for energy acquisition, microbial protein synthesis, and further energy deposition within the body. Although different putative functional characteristics were observed, their similar *in vitro* fermentation responses and close clustering in PCoA distance would suggest that the two isolates may modulate and improve fermentation reactions associated with degradation in a similar way, but different from the case of isolate B. Production of high-protein milk cow microbiota enhanced functional potential related to branched-chain amino acid biosynthesis (57), which is partly consistent with the predicted pathways driven by isolates 11 and 14. This suggests its potential applications in higher production efficiency through future *in vivo* study.

### 4.3 Limitations and future perspectives

The effects of probiotic supplementation on nutrient utilization efficiency depend largely on the ration design, and the observed incubation time results may not represent the responses of production cattle fed higher proportions of concentrate. The lack of a marked impact on digestibility or bacterial abundance may be due to the relatively limited incubation period, during which the supplemented isolates were not fully established within the rumen microbial community (49). The 24 h incubation period may not have been long enough to capture the full influence of the isolates on *in vitro* fermentation. The effects of microbial supplementation may depend on the incubation duration and may become more evident over longer periods (49, 58).

However, the present study successfully provided a preliminary understanding of how goat rumen-derived isolates affected *in vitro* rumen microbial community. Our findings warrant comprehensive research approaches, including *in vivo* feeding trials and integrated methodologies, such as metabolomic and proteomic analyses, together with microbial interaction network analysis (14), to clarify the practical effects of these isolates and their influence on livestock productivity. Our results suggest that these rumen-derived goat isolates have multiple potential applications particularly for younger animals. A compound probiotic containing strains belonging to *Lactobacillus, Pediococcus, and Bacillus* altered rumen fermentation, promoted rumen development, and improved health-related indicators (59). The in vivo effects of the mixed isolate supplementation on different type of animals warrant further investigation.

## 5 Conclusion

Different goat rumen-derived isolates can modulate *in vitro* fermentation and microbial communities and their predicted functions. In particular, isolate B (*Pediococcus* sp.) substantially decreased *in vitro* rumen pH and increased total VFA accumulation, indicating a shift toward soluble carbohydrate utilization and enrichment in sugar-related metabolism. Isolates 11 (*Enterococcus* sp.) and 14 (*Streptococcus* sp.) were mainly associated with enhanced fermentation activity and were enriched in fiber-metabolizing bacteria. It is possible that these isolates influence microbial communities toward more active substrate utilization and fermentative metabolism, thereby contributing to enhanced rumen function by inhabiting microbes.

## Data Availability

The datasets presented in this study can be found in online repositories. The names of the repository/repositories and accession number(s) can be found in the article/Supplementary material.
