# Supplementary material for: Characterization of goat rumen-derived isolates and their effects on in vitro ruminal fermentation properties
Source: Front Vet Sci. 2026 Jul 3;13:1877756. doi: 10.3389/fvets.2026.1877756 (PMC13377694; doi:10.3389/fvets.2026.1877756)
Supplement: Supplementary file 1 [file Data_sheet_1.docx]

Characterization of goat rumen derived isolates and their effects on *in vitro* ruminal fermentation properties

Zhang Yushu,^1^ and Y. Uyeno^1*^

^2^ Graduate School of Science and Technology, Shinshu University, Nagano, Japan

**Supplementary table and figure**

| Supplementary table 1. Representative closest BLAST matches of the isolates based on 16S rRNA gene sequences and conservative taxonomic assignment | | | |
| --- | --- | --- | --- |
| Isolates | Closest relative (accession no.) | Similarity (%) | Assigned taxon |
| Isolate 11 | Enterococcus faecium strain GG2 (KX426269.1) | 99.93 | *Enterococcus* sp. |
| Isolate 14 | Streptococcus equinus strain 2B (KF156794.1) | 99.86 | *Streptococcus* sp. |
| Isolate 7 | Streptococcus equinus strain CNU 13 (MN075421.1) | 99.86 | *Streptococcus* sp. |
| Isolate B | Pediococcus acidilactici strain JFP1 (KM062019.1) | 99.73 | Pediococcus sp. |

| Supplementary table 2 Effects of inoculation dose linear and quadratic effect on in vitro rumen fermentation parameters | | | | | | | | | | | |
| --- | --- | --- | --- | --- | --- | --- | --- | --- | --- | --- | --- |
|  | Bacteria B | |  | Bacteria 11 | |  | Bacteria 14 | |  | Bacteria 7 | |
|  | Linear | Quadratic |  | Linear | Quadratic |  | Linear | Quadratic |  | Linear | Quadratic |
| pH | 0.032 | 0.888 |  | 0.881 | 0.797 |  | 0.139 | 0.034 |  | 0.305 | 0.026 |
| 24h Gas Production (mL/g) | 0.015 | 0.991 |  | 0.959 | 0.245 |  | 0.031 | 0.239 |  | 0.063 | 0.015 |
| Methane % | 0.076 | 0.877 |  | 0.417 | 0.519 |  | 0.162 | 0.008 |  | 0.440 | 0.108 |
| Methane (mL/g) | 0.011 | 0.947 |  | 0.655 | 0.797 |  | 0.234 | 0.070 |  | 0.480 | 0.019 |
| Digestibility% | 0.265 | 0.908 |  | 0.126 | 0.112 |  | 0.847 | 0.600 |  | 0.004 | 0.077 |
| AA% | 0.160 | 0.399 |  | 0.194 | 0.108 |  | 0.198 | 0.741 |  | 0.220 | 0.232 |
| PA% | 0.196 | 0.317 |  | 0.299 | 0.107 |  | 0.303 | 0.875 |  | 0.375 | 0.308 |
| BA% | 0.475 | 0.355 |  | 0.424 | 0.462 |  | 0.485 | 0.416 |  | 0.816 | 0.718 |
| Total VFA (mmol/L) | 0.067 | 0.896 |  | 0.962 | 0.960 |  | 0.166 | 0.103 |  | 0.689 | 0.516 |
| A/P | 0.176 | 0.335 |  | 0.262 | 0.102 |  | 0.267 | 0.863 |  | 0.325 | 0.274 |
| Bactria (10^7^ copies/mL) | 0.359 | 0.130 |  | 0.750 | 0.056 |  | 0.279 | 0.824 |  | 0.158 | 0.393 |
| Archaea (10^7^ copies/mL) | 0.094 | 0.596 |  | 0.479 | 0.130 |  | 0.347 | 0.659 |  | 0.274 | 0.689 |
| NH_3_-N (mg/100 mL) | 0.960 | 0.586 |  | 0.570 | 0.665 |  | 0.512 | 0.190 |  | 0.871 | 0.493 |

| Supplementary table 3. Pairwise PERMANOVA results based on Bray–Curtis distances among treatment groups | | | | |
| --- | --- | --- | --- | --- |
| Group1 | Group2 | R^2^ | P value | P adjust |
| Control | Isolate 11 | 0.437 | 0.100 | 0.111 |
| Control | Isolate 14 | 0.412 | 0.100 | 0.111 |
| Control | Isolate 7 | 0.642 | 0.100 | 0.111 |
| Control | Isolate B | 0.634 | 0.100 | 0.111 |
| Isolate 11 | Isolate 14 | 0.218 | 0.300 | 0.300 |
| Isolate 11 | Isolate 7 | 0.650 | 0.100 | 0.111 |
| Isolate 11 | Isolate B | 0.669 | 0.100 | 0.111 |
| Isolate 14 | Isolate 7 | 0.575 | 0.100 | 0.111 |
| Isolate 14 | Isolate B | 0.592 | 0.100 | 0.111 |
| Isolate 7 | Isolate B | 0.293 | 0.100 | 0.111 |

| Supplementary table 4. Information on Japanese Shiba goats used for the experiment. | | | |
| --- | --- | --- | --- |
| Goat ID | Sex | Date of birth | Health status |
| Goat 1 | Female | 2016.12.20 | Healthy |
| Goat 2 | Female | 2016.12.20 | Healthy |
| Goat 3 | Castrated male | 2020.04.29 | Healthy |
| Goat 4 | Castrated male | 2020.04.29 | Healthy |


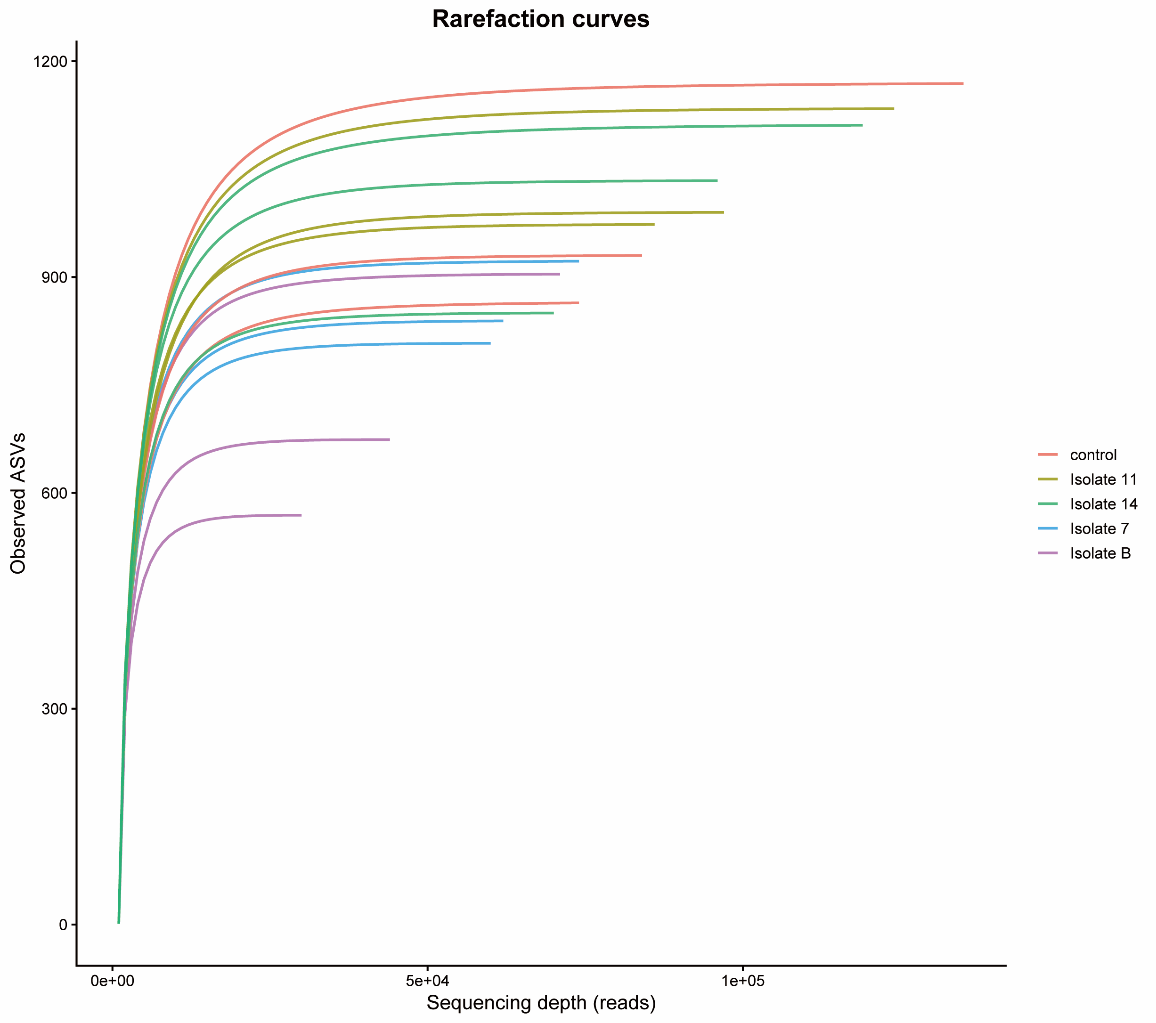


Supplementary Fig.1. Rarefaction curve of observed ASVs across treatments


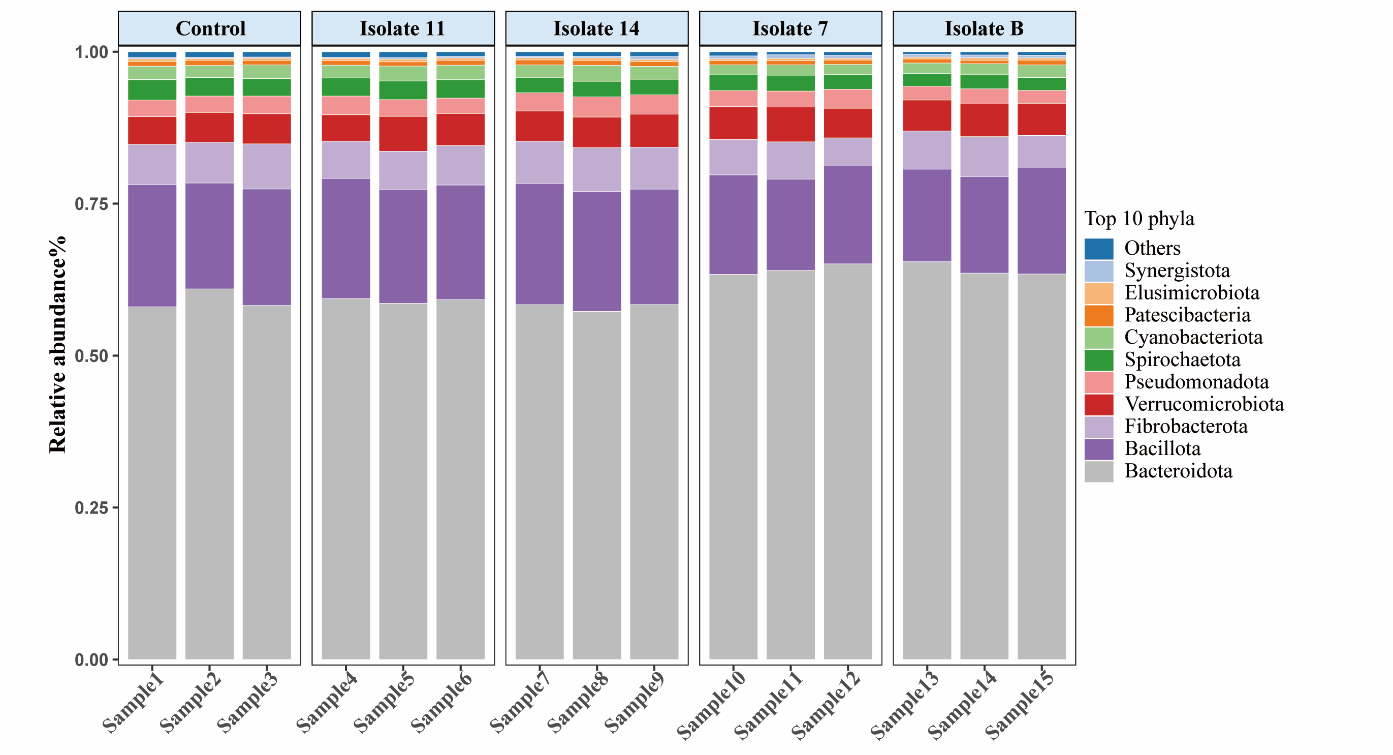


Supplementary Fig.2. Relative abundance of bacterial communities at the phylum level


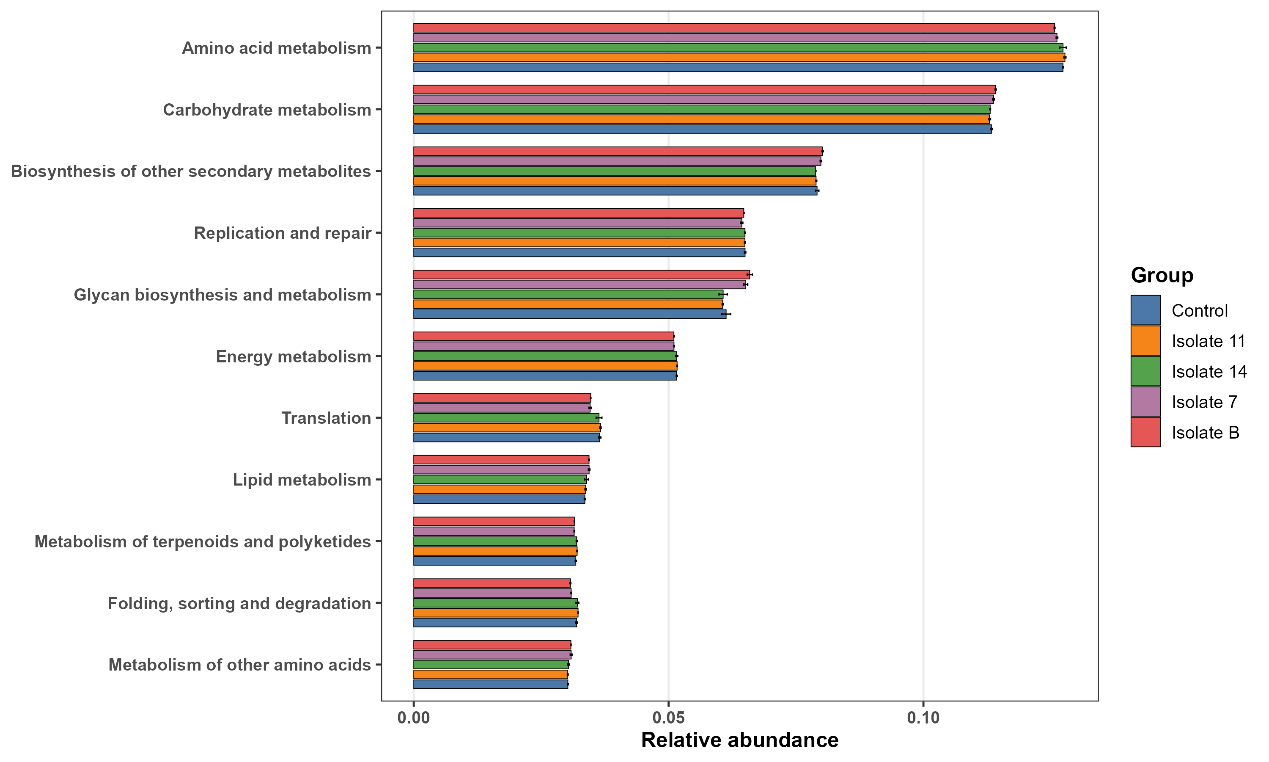


Supplementary Fig.3. Relative abundance of major predicted KEGG level 2 functional categories across treatment groups





Supplementary Fig.4. Correlation heatmap between 24 h culture parameters and growth in pure cultures of different rumen-derived isolates.
